# Supplementary material for: Association of center-level operative volume and acute outcomes following robotic-assisted colectomy for colorectal cancer
Source: PLoS One. 2025 Jun 25;20(6):e0299174. doi: 10.1371/journal.pone.0299174 (PMC12193071; doi:10.1371/journal.pone.0299174)
Supplement: S1 Table — (DOCX) [file pone.0299174.s001.docx]

|  | ICD-10 Diagnosis Codes |
| --- | --- |
| Colorectal Neoplasm (Benign or Malignant) | C18, C19, C20, D12 |
| Conversion to Open Procedure | Z53.31 |
|  | ICD-10 Procedure Codes |
| Robotic Operations | 8E0W0CZ, 8E0W3CZ, 8E0W4CZ, 8E0W7CZ, 8E0W8CZ, 8E0WXCZ, 8E0X0CZ, 8E0X3CZ, 8E0X4CZ, 8E0XXCZ, 8E0Y0CZ, 8E0Y3CZ, 8E0Y4CZ, 8E0YXCZ |
| Colectomy | 0DTF4ZZ, 0DTH4ZZ, 0DTK4ZZ, 0DBF4ZZ, 0DBH4ZZ, 0DBK4ZZ, 0DTF0ZZ, 0DTH0ZZ, 0DTK0ZZ, 0DBF0ZZ, 0DBH0ZZ, 0DBK0ZZ, 0DTL4ZZ, 0DBL4ZZ, 0DTL0ZZ, 0DBL0ZZ, 0DTM4ZZ, 0DTG4ZZ, 0DBM4ZZ, 0DBG4ZZ, 0DTM0ZZ, 0DTG0ZZ, 0DBM0ZZ, 0DBG0ZZ, 0DTN4ZZ, 0DBN4ZZ, 0DTN0ZZ, 0DBN0ZZ, 0DTE4ZZ, 0DBE4ZZ, 0DTE0ZZ, 0DBE0ZZ, 0D1B0ZQ, 0D1B8ZQ, 0D1B4ZQ |
| Proctectomy | 0DTP0ZZ, 0DTP4ZZ |

**Supplemental Table S1**. International Classification of Disease, Ninth and Tenth revision, Codes (ICD-9/10) for Robotic Operations and Colectomy Procedures.
