# Supplementary material for: Association of center-level operative volume and acute outcomes following robotic-assisted colectomy for colorectal cancer
Source: PLoS One. 2025 Jun 25;20(6):e0299174. doi: 10.1371/journal.pone.0299174 (PMC12193071; doi:10.1371/journal.pone.0299174)
Supplement: Attachment — Submitted filename: Point-by-Point_Cover Letter.doc [file pone.0299174.s003.doc]

**David Geffen School of Medicine at UCLA**

**Ronald Reagan-UCLA Medical Center**

Department of Surgery

Division of Cardiac Surgery

David Geffen School of Medicine at UCLA

62-249 Center for Health Sciences

Los Angeles, CA 90095-1741

Tel: 310-206-6717

Email: PBenharash@mednet.ucla.edu

PEYMAN BENHARASH, MD

Associate Professor of Surgery and Bioengineering

Associate Director Thoracic Residency Program

Director of Adult ECMO Program at UCLA

Director of UCLA Cardiac Outcomes Research Laboratories

September 15, 2024

**RE: Revisions for manuscript entitled “Association of Center-Level Operative Volume and Acute Outcomes following Robotic-Assisted Colectomy for Colorectal Cancer”**

Dear Dr. Chenette and the esteemed members of the Editorial Board,

On behalf of my co-authors, I would like to thank the reviewers for their thorough review of our manuscript. We deeply appreciate the profound and nuanced comments made by the reviewers and hope that we have adequately addressed them. Please find our point-by-point responses below. The pertinent modifications are tracked and edited in the revised manuscript.

We express our sincerest appreciation to the editorial team and reviewers for the providing us the opportunity to revise the manuscript titled above for potential publication in *PLOS One*. We look forward to your team’s thorough review of our manuscript and would be happy to provide any further information as deemed necessary. Thank you for your invaluable time and your thoughtful consideration.

Sincerely,

Peyman Benharash, MD

**Reviewer #1: This is a retrospective review using the Nationwide Readmission Database looking at costs and major adverse events with the use of the robot for colectomy based on volume.**

**I was wondering if this database covers years current (such as 2020-2023)? If so, why were they not included.**

Thank you for this comment. Recent years were unavailable due to the data collection and processing time required by Agency for Healthcare Research and Quality.

**It was really unclear how costs were calculated in this study-making the conclusions difficult to interpret. I am not sure that the Nationwide Readmissions database is the most reliable database to use for this analysis. Especially since only a little over 50% of hospitalizations have accurate information.**

Thank you for your comment. The Nationwide Readmission Database (NRD) is the largest database that tracks inpatient hospitalization costs, using discharge data from 30 geographically dispersed states, representing 59.6% of all U.S. hospitalizations. While some bias may occur due to coding errors, the NRD is widely regarded as a reliable and accurate data source for the hospitalizations it includes. By comparison, the National Inpatient Sample (NIS) provides cost information but only represents a 20-percent stratified sample of all discharges from U.S. hospitals. This makes the NRD’s 59.6% coverage a more accurate and comprehensive sample.

**In the methodology it indicates that both benign and malignant neoplasms data was pulled from, but the entire manuscript indicates malignancy.**

We appreciate this comment. Manuscript wordings have been modified accordingly.

**Why were only colectomies included and not proctectomies? That is where the real benefit of the robot of colorectal surgery has been seen.**

Thank you for your comment. In our database, we identified ~1000 patients undergoing robotic proctectomy for benign or malignant neoplasms of the colon, sigmoid junction, or rectum each year. Due to the relatively low volume of robotic prostatectomies, it was likely that these procedures were concentrated in highly specialized centers with higher surgical volumes. As a result, they were not a primary focus of our study.

**I don’t think calculating high versus low based on overall robotic operations at the institution each year is appropriate. Given that most of the procedures at LVH were not elective I don’t think looking at costs or MAE is accurate given these are usually sicker patients.**

Thank you for this important point. To ensure a better comparison group, we narrowed our analysis to elective operations only. Following the exclusion of non-elective patients, management at LVH was associated with greater odds of MAE and incremental cost.

**Don’t understand the statement of prehab and rates of liver mets- not accurate and not supported by that reference.**

We appreciate your point. Given that studies looking into prehabilitation with a reduction in hospitalization costs for cancer patients undergoing robotic surgeries are sparse in the literature and not supported by the data presented, we removed the sentence. We have added the following line to our limitation:

“We also could not assess the institutional capacity to monitor and provide prehabilitation or enhanced recovery pathways after surgery.” (Page 15)

**Reviewer #2:**

**1. When you use an abbreviation in the text for the first time, it is recommended to write the full name and follow it immediately with an abbreviation. RAC in the abstract section.**

Thank you for this comment. We have revised our abstract accordingly.

**2. How were high and low-volume hospitals defined in your study?**

We appreciate your comment. We have added the following lines to the method section to further clarify our definition of high- and low- volume centers.

“We utilized institutional robotic-assisted colectomy volume as a continuous variable in the spline analysis to evaluate the relationship between procedural volume and MAE.

To identify the inflection point, we calculated the global maximum of the second derivative in the spline between volume and MAE. Institutions with volumes at or above this inflection point (N=207) were classified as high-volume hospitals (HVHs), while those below the threshold were designated as low-volume hospitals (LVHs).” (Page 6)

**3. Please revise your reference on the costs of colectomy surgery in the introduction section (the cost of colectomy open surgery nearly doubled that of laparoscopic and robotic surgery?)**

Thank you very much for this point. We have added the following reference to the manuscript [1]. Similar to their study, we found open operations were approximately 120% of the cost of laparoscopic and robotic approaches.

1.	Simianu VV, Gaertner WB, Kuntz K, et al. Cost-effectiveness Evaluation of Laparoscopic Versus Robotic Minimally Invasive Colectomy. Ann Surg. 2020;272(2):334. doi:10.1097/SLA.0000000000003196

**4. You mentioned in your study that the operating room level may contribute to low cost, I suggest you write this point more clearly for your readers to understand.**

We appreciate your comment. We have edited our discussion section to further elaborate on operating room level interventions that may contribute to lower cost.

“Notably, improving operating room turnover efficiency and fostering comprehensive operating room staff training to enhance proficiency with the robotic platform are critical measures to lower costs. Current cost reduction strategies primarily focus on minimizing instrument redundancies and enhancing operative efficiency, both of which are facilitated by increasing surgeon proficiency through training and experience.24,25 In order to develop a more cost-effective framework for robotic-assisted operations, further investigation on driving factors of cost in RAC is warranted.” (Page 13).

**5. You seem to indicate that prehabilitation protocols contribute to cost reduction, can you elaborate more?**

Thank you for this comment. Previous studies have shown that the prehabilitation pathway, with or without enhanced recovery after surgery protocol, may reduce the risk of bleeding complications, hospital duration of stay, and cost. [1] In patients with cancer, prehabilitation improves functional outcomes in the patients after cancer resection operations [2]. Nonetheless, given our database is limited to inpatient data, we removed the sentence regarding cost reduction as our data cannot support the claim.

1.	Ploussard G, Almeras C, Beauval J, et al. A combination of enhanced recovery after surgery and prehabilitation pathways improves perioperative outcomes and costs for robotic radical prostatectomy. Cancer. 2020;126(18):4148-4155. doi:10.1002/cncr.33061

2.	Coderre D, Brahmbhatt P, Hunter TL, Baima J. Cancer prehabilitation in practice: the current evidence. Curr Oncol Rep. 2022;24(11):1569-1577. doi:10.1007/s11912-022-01304-1

**Reviewer #3:**

**According to the authors, the Nationwide Readmissions Database (NRD), a component of the Nationwide Healthcare Cost & Utilization Project (HCUP) run by the Agency for Healthcare Research and Quality (AHRQ), is where the data used to support the study’s conclusions may be found. However, researchers who meet the requirements for access to secret data must complete a Data Use Agreement in order to access this data. The authors cannot immediately disclose the data because they require explicit consent from AHRQ, and they do not have any special access credentials that others would not have. As a result, even while the data is available, there are limitations that prevent complete access.**

We appreciate this comment. We will make sure to acknowledge this point when we submit the manuscript.

**Another statistical test that could be used in addition to cross-volume analysis is multivariable logistic regression. This test is particularly useful for examining the relationship between one or more independent variables (such as institutional robotic surgery volume, patient demographics, and comorbidities) and a binary dependent variable (such as the occurrence of major adverse events or readmissions).**

**Thus the use of ICT could enhance the data collection and thus it could make the research in ease and more variables could be identified.**

Thank you very much for this comment. We have used multivariable logistic and linear regression to evaluate the association of HVH status with outcomes of interest as suggested.
